# Innovative Protein Ingredients for Feeding Gilthead Seabream (*Sparus aurata*) Broodstock

**DOI:** 10.1155/anu/4229257

**Published:** 2025-06-29

**Authors:** Lucia Aidos, Giorgio Mirra, Mirko Sergio, Margherita Pallaoro, Maria Chiara Di Meo, Chiara Cialini, Chiara Bazzocchi, Silvia Clotilde Bianca Modina, Lorenzo Proietti, Luciano Foglio, Francisco Javier Alarcón-López, Katia Parati, Alessia Di Giancamillo

**Affiliations:** ^1^Department of Veterinary Medicine and Animal Sciences, University of Milan, Via dell'Università 6, Lodi 26900, Italy; ^2^Department of Biomedical Sciences for Health, University of Milan, Via Mangiagalli 31, Milan 20133, Italy; ^3^Department of Sciences and Technologies, University of Sannio, Benevento 82100, Italy; ^4^The Italian Experimental Institute “Lazzaro Spallanzani”, Rivolta d'Adda, Italy; ^5^Department of Biology and Geology, University of Almeria, Almería 04120, Spain

**Keywords:** morphology, myogenesis, skeletal development, soy replacement, *Sparus aurata*

## Abstract

A feeding trial with alternative protein sources was conducted in gilthead seabream (*Sparus aurata*, L.) broodstock fed a control diet and two diets with 5% or 10% inclusion levels of a blend of novel ingredients. The blend is composed of 40% insect (*Hermetia illucens*), 10% duckweed (*Lemna minor*), 45% *Nannochloropsis gaditana*, and 5% *Alaria esculenta* meal dry biomass. The animals were reared in a recirculating aquaculture system and administered the experimental diets 3 months before spawning and for a total of 7 months. Fertilized eggs were incubated until hatching and newly hatched larvae were monitored until the yolk sac absorption stage. High inclusion (HI; 10%) of novel ingredients to broodstock resulted in a significantly higher hatching rate, while both groups fed novel ingredients produced offspring with significantly higher survival until the end of the yolk sac stage compared to offspring from parents fed the control diet (*p* < 0.05). The inclusion of the protein blend at any level did not produce differences in larval growth. Morphological and histometric analyses in larvae revealed hypertrophic growth during the yolk sac stage. The expression of genes involved in muscle development and growth indicated no differences in growth potential in larvae between groups. Overall, broodstock feeds for gilthead seabream can have an inclusion of novel ingredients without a negative impact on larval performance and growth. Further studies are needed to study the long-term effects of broodstock diet on offspring quality.

## 1. Introduction

Carnivorous fish generally have higher dietary protein requirements than omnivorous and herbivorous species [1, 2]. Farmed carnivorous species' dietary protein levels vary between 40% and 55% to achieve maximum growth [3]. Breeders of carnivorous marine fish like seabream (*Sparus auratus*) and seabass (*Dicentrarchus labrax*) require an even higher amount of raw protein (50%–55%) [4]. The reason for this is that protein is the most abundant nutrient in fish eggs [5], responsible for the embryo's growth and, in most teleost species, it constitutes the main energy source during embryonic development [4, 6]. Additionally, fish meal (FM), the main protein source of traditional aquafeeds, not only provides an optimal amino acid profile but may also contain essential factors that could be critical during embryogenesis and larval growth, particularly in sustaining maximum health and performance in offspring. As reviewed by Fernández-Palacios et al. [4], several studies showed that gilthead seabream broodstock dietary protein with a balanced composition of amino acids is vital for the embryo, as it improves the synthesis of vitellogenins.

In the last decades, the aquaculture sector has been searching for alternatives to FM, since it is derived from marine sources and its availability is subject to natural and economic limitations. However, FM remains an industry standard in broodstock feeds due to its precise mix of primary nutrients, critical for reproduction. Despite this, the aquaculture industry has focused its efforts on finding FM substitutes, predominantly favoring vegetable feedstuffs [7]. However, these alternatives often have lower nutritional values [8]. The most common plant ingredient in fish feed is soybean meal (SBM), as it represents a good alternative to FM due to its high protein content, low price, and good availability. However, its amino acid profile is not adequate, often lacking in methionine [9], and it contains antinutritional factors that induce gut microbiome dysbiosis and inflammatory responses, for example, SBM-induced enteritis [9, 10]. Other plant ingredients widely used in fish feed are corn and wheat gluten, but even in this case, the amino acid profile is unbalanced compared to the nutritional needs of carnivorous fish, with deficiencies in essential amino acids [11]. Too high inclusions (HIs) of these traditional vegetable flours, in the aquafeed of carnivorous fish, is not possible as it would lead to reduced growth, harmed digestive activity, lipid accumulation in the liver, and oxidative stress [12, 13] with the downregulation of protein biosynthesis-related genes in liver [14]. Furthermore, the massive use of traditional vegetable ingredients also used in human food and the environmental issues due to soy cultivation, such as biodiversity loss, carbon emission, and land depletion, have led to a focus on finding alternative protein sources that have less impact on fish health and the environment. Alternative protein sources like microalgae, yeast, bacteria, macroalgae, and insect meal are increasingly used and constitute sustainable ingredients [15–19] (UE recommendation, AAC 2023), offer balanced amino acids and omega-3s [20] and contain bioactive compounds that enhance digestion, immunity, stress tolerance, and growth [15, 16, 21]. Given the potential of alternative proteins and the importance of broodstock nutrition on offspring quality, this study evaluated the effects of partially replacing traditional vegetable ingredients with alternative protein sources (*Alaria esculenta*, *Nannochloropsis gaditana*, *Lemna minor*, *and Hermetia illucens*) in gilthead seabream feed, assessing impacts on both broodstock fish and larvae. All those alternative ingredients, algae, insect larvae, and duckweed have been selected as promising alternatives to traditional feedstuffs such as soybean and rapeseed meal, FM, or oilseeds due to their high protein or fat contents, along with a high abundance of vitamins, minerals, and bioactive compounds. *Nannochloropsis gaditana* is characterized by a significant amount of crude protein, typically ranging from 30% to 55% on a dry weight basis with an adequate content of essential amino acids, especially in lysine (0.51%) and methionine (1.8%), which are widely acknowledged as the most limiting amino acids in ingredients for aquafeeds [22]. Furthermore, this microalgae species is able to accumulate up to 48% lipid content in dry mass, enriched in eicosapentaenoic acid (EPA; 20:5*n*−3) [23]. The potential of this microalga as a feed ingredient has been previously demonstrated when a low dietary inclusion level (5%) is used for feeding juvenile gilthead seabream [24]. *Alaria esculenta* has a modest content of protein but is a natural source of bioactive substances, such as fucoxanthin, which represents approximately 4.1% of its total lipids. Indeed, noticeable anti-inflammatory and antioxidant activities have been reported for this species [25]. Duckweed (*L. minor*) contains a wide variety of compounds (β-carotene, lutein, lycopene, and neoxanthin) which have shown effects as immunonutrients, antioxidants, or modulators of intestinal microbiota, mainly in freshwater fish species [26]. However, the presence of some antinutrient compounds has been described in duckweed, which could limit its dietary inclusion level, particularly in the case of marine fish where scarce information about its application is available [27]. *Hermetia illucens* larvae meal has a high content of a well-balanced amino acid profile and has received much attention as an alternative protein source to replace fishmeal in aquafeeds [28].

In fish, muscle growth is regulated by myogenic regulatory factors (MRFs), including myogenic factor 5 (*myf5*), myoblast determination protein (*myod*), myogenic factor 6 (*mrf4*), and myogenin (*myog*). These factors orchestrate the formation and differentiation of two main types of muscle fibers: slow-twitch (red fibers), which support endurance and sustained activity, and fast-twitch (white fibers), which are used for quick and powerful movements. *myf5* and *myod* (early MRFs) lead the creation of new muscle fibers, while MRF4 and myogenin (late MRFs) further guide these fibers' differentiation and maturation, ensuring proper development. Fish muscle growth follows the mosaic muscle development, in which newly formed fibers are continuously integrated into the muscle, alongside preexisting fibers, throughout the fish's life. This allows for continuous growth and adaptation, particularly in response to environmental demands.

The focus of this study was to assess the effect of novel ingredients in diets on broodstock gilthead seabream as a partial replacement for the classical vegetable ones. A blend of novel ingredients with potential functional properties was formulated, dosing the nutritional contributions of each of them to obtain a balanced mix. The blend was composed of 40% *H. illlucens*, 45% *N. gaditana* as major components for providing protein, EPA, and bioactive compounds, 10% *L. minor*, and 5% *A. esculenta* as sources of bioactive compounds. The blend was used at two different dietary inclusions (5% and 10%), to study in vivo their effects on the broodfish in terms of reproductive parameters and the indirect effect of the diet on the growth and muscle development of larvae, from hatching to yolk sac resorption.

## 2. Material and Methods

### 2.1. Novel Ingredients

The blend of alternative ingredients was composed of insect meal obtained for *H. illucens* larvae (56.5% crude protein and 15.2% crude lipid) provided by Leibniz-Institut fur Agrartechnik (Germany), duckweed meal (*L. minor*) (33.5% crude protein and 6.6% crude lipid) provided by Institute of Technology of Agricultural Products (Greece), microalgae meal (*Nannocloropsis gaditana*; 31.4% crude protein and 21.3% crude lipid) provided by University of Almería (Spain), and macroalgae meal (*A. esculenta*; 17.2% crude protein and 2.7% crude lipid) provided by Bantry Marine Research Station (Ireland). The fatty acids and amino acids profiles of the alternative ingredients are detailed in Table 1. The blend was composed of 40% *H. illlucens*, 45% *N. gaditana* as major components for providing mainly protein and EPA and also bioactive compounds, and 10% and 5%, of *L. minor* and *A. esculenta*, respectively. The contribution of those last ingredients to the blend was lower owing to they are considered mainly as sources of bioactive compounds instead of macronutrients. With the exception of the insect meal, the other alternative ingredients were previously enzymatically hydrolyzed. Briefly, for the enzymatic hydrolysis, alternative ingredients were suspended (100 g dry weight/L) in 50 mM sodium citrate buffer (pH 5.0) and incubated at 40°C under continuous agitation for 4 h in presence of a blend of carbohydrases (xylanase 20,000 U/g; glucanase; 30,000 U/g; cellulase 10,000 U/g, and protease 10,000 U/g) providing a 0.05 enzyme to algae ([E]/[S]) ratio. Immediately after the hydrolysis, the reaction mixture was heated at 80°C for 15 min in order to inactivate the enzymes. The experimental diet formulation and preparation were carried out by the Experimental Diets Service of CEIMAR-University of Almeria (Almeria, Spain), using standard procedures for aquaculture feed processing. Briefly, all ingredients were mixed in a 120 L mixer and ground with a hammer mill (UPZ 100, Hosokawa-Alpine, Augsburg, Germany) until 0.5 mm particles were obtained. Subsequently, the diets were extruded using a twin-screw extruder (Evolum 25, Clextral, Firminy, France) equipped with dies suitable for the production of 8 mm sinking pellets. The extruder barrel consisted of four sections with temperature profiles of 100, 95, 95, 95, and 90°C, respectively, along the sections. After extrusion, the pellets were dried at 30°C in a 12 m^3^ drying chamber with forced air circulation (Airfrio, Almeria) for 12 h, and then, cooled to room temperature. Vacuum oil coating was done on the following day in a Pegasus PG-10VC LAB vacuum coater (Dinnissen, The Netherlands). Then, feeds were kept in sealed plastic bags at −20°C until use.

### 2.2. Experimental Diets

A blend of the novel ingredient composed of 40% insect (*H. illucens*), 10% duckweed (*L. minor*), 45% *Nannochloropsis gaditana*, and 5% *A. esculenta* meal dry biomasses were included in two experimental diet at 5% and 10% inclusion, low inclusion (LI) and HI, respectively. The fatty acid and amino acid profiles, the ingredients, and the nutrient composition of the experimental diets are detailed in Tables 2–4. All diets were formulated to be isoenergetic (18 MJ/kg), isoprotein (55%), and isolipid (18%).

### 2.3. Experimental Set-Up and Rearing Conditions

The trial was conducted at the experimental facilities of Istituto Sperimentale Italiano “Lazzaro Spallanzani”, Rivolta d'Adda, Italy. Reproducers of gilthead seabream (*Sparus aurata*) were obtained from a broodstock breed from Maricoltura farm, in Rosignano Solvay. After arrival at the experimental unit, fish were kept under quarantine conditions and once they were acclimated to the new rearing facilities, the specimens were randomly assigned to three feeding dietary groups. For each dietary group, there were three replicates (25 fish/replicate, at a female–male ratio of 1:2); each replicate consisted of a tank of 5 m^3^ capacity. Two groups were fed two experimental diets containing 5% and 10% of the alternative blend of alternative ingredients and a control group was fed a control diet mimicking the composition of commercial aquafeed for gilthead seabream broodstock (Table 1). The feeding trial was carried out on the fish's final reproductive phase and lasted for 7 months.

The initial body weight means values ±SEM of the females was 252 ± 35 g and 510 ± 90 g of the males, and the initial stocking density was 5.07 kg/m^3^.

All the tanks were supplied with seawater (38 g/L salinity, pH 8), and maintained under natural photoperiod. The saltwater has been reconstituted using a high-quality sea salt, containing all the trace minerals originally present in the seawater (Ocean Fish—Prodac). The daily water change was about 2%. The water temperature in the tanks was regulated using a heat pump and maintained at 18–20°C. An adequate oxygenation level was maintained with aerators (above 6 mg/L).

The fish handling procedures and sampling methods used in the trial adhered to the E.U. directive 2010/63/EU guidelines on the protection of animals used for scientific purposes (The AquaTech4Feed ID project:143, Work Package 3).

### 2.4. Reproductive Parameters

Spawning performance and egg quality were controlled daily, over 123 days, from the middle of January (when all the broodstock was synchronized with the spawning) until the end of May. A passive egg collector was placed in the outflow of each spawning tank, to collect the spawned eggs. Spawned eggs, collected from each tank, were collected every morning (~18 h after spawning) into a 10-l bucket, provided with aeration for sample homogenization. From this pool of eggs, five randomized 5-mL samples were taken and placed in a Bogorov chamber, under a stereoscope to calculate the total number of eggs (fecundity parameter) and percentages of fertilized, unfertilized, and viable eggs. The fecundity parameter was estimated by counting the total number of eggs in a subsample of 5 mL, by using a Bogorov chamber. Fertilization success was evaluated simultaneously by examining the sampled eggs for the presence of viable embryos (usually at the blastula stage).

Eggs were then transferred to 24-well microtiter plates, according to the incubation set-up performed by Chatzifotis et al. [30]: viable eggs were placed individually in each well, with 2.5 mL of sterilized seawater at the same salinity as the rearing tanks. A steady temperature of 18/19°C was maintained. The water of every well was monitored for the classic quality parameters until the end of the yolk sac absorption.

The plates were kept in a semi-dark room and were checked, following the procedure developed by Panini et al. [31]. The plates were evaluated daily to verify: the development stage, number of hatched larvae, and number of viable larvae until the yolk sac was absorbed. Hatching and Larval survival rates were calculated on a subsample of 38 egg lots randomly sampled from the total eggs collected from January to May. The hatching rate was calculated as the number of hatched larvae divided by the total number of fertilized eggs. Larval survival at the end of the yolk sac absorption was examined under a stereomicroscope and calculated as the number of live larvae at the end of the yolk sac absorption/number of hatched larvae.

### 2.5. Larvae

#### 2.5.1. Sampling

At hatching six larvae were sampled from each replicate (18 larvae collected per experimental group), while the other newly hatched larvae were kept in the wells; at the complete yolk sac absorption stage (t1), six larvae were sampled from each replicate (18 larvae collected per experimental group). For each time point, a total of 54 larvae were sampled. All sampled larvae were euthanized with an overdose of the anesthetic, 500 mg/L of ethyl 3-aminobenzoate, methanesulfonic A (Sigma–Aldrich, Milan, Italy). For morphological analyses, larvae sampled at both timepoints, were fixed in neutral formalin and kept at 4°C and others were frozen in liquid nitrogen and stored at −80 °C for subsequent molecular analyses.

#### 2.5.2. Zootechnical Data

Larvae from the three experimental groups (four larvae per group) were measured from head to tail at both hatching and the end of the yolk sac absorption. All measurements were performed using the image software Optika Proview software (Optika, Italy).

#### 2.5.3. Skeletal Development—Whole-Mount Histochemical Staining

Alcian blue–Alizarin red whole-mount skeletal staining allows the evaluation of the skeletal elements and respective locations. The double staining applied to formalin-fixed larvae used in this study was performed according to Rigueur and Lyons [32]. Briefly, larvae were dehydrated in a graded ethanol series and incubated in an Alcian Blue 8GX solution for 2 h at room temperature. Larvae were then rehydrated in a graded ethanol series and incubated for 1 h at room temperature, in an Alizarin Red solution (1 mg/mL). Following, larvae were washed three times for 3 min in distilled water and were placed in a graded series of glycerol solutions (20%, 40%, 70%, and 100%). Subsequently, larvae were visualized and documented using an optical Olympus BX51 light microscope (Olympus, Opera Zerbo, Milan, Italy) equipped with a digital camera.

#### 2.5.4. Muscle Development—Whole-Mount Immunofluorescence and Histometry

Muscle development was assessed by anti-rabbit skeletal muscle actin antibody (ACTA1), whole-mount immunofluorescence. Formalin-fixed larvae were used and the whole mount staining was performed as described in [33]. Briefly, larvae were washed in running water and incubated with the first-step primary antiserum mouse monoclonal anti-α-Actin (Invitrogen, MA5-14084), 1:25 in PBST for 24 h at room temperature, then, washed three times in phosphate-buffered saline (PBS) for 5 min and incubated with a solution of 10 µg/mL goat biotinylated anti-rabbit IgG (Vector Laboratories Inc., Burlingame, CA, USA) for 3 h at room temperature. Larvae were then washed twice in PBS and treated with Fluorescein-Avidin D (Vector Laboratories Inc. Burlingame, CA, USA) 10 µg/mL in NaHCO_3_, 0.1 M, pH 8.5, 0.15 M NaCl for 2 h, at room temperature. Finally, after washing three times in PBE, larvae were embedded in Vectashield Mounting Medium with DAPI (40,6-diamidino-2-phenylindole; H-1200, Vector Laboratories Inc., Burlingame, CA, USA) and observed using a confocal laser scanning microscope (FluoView FV300; Olympus, Segrate, Italy). The immunofluororeactive structures were excited using Argon/Helio–Neon–Green lasers with excitation and barrier filters set for fluorescein. Images containing superimposition of fluorescence were obtained by sequentially acquiring the image slice of each laser excitation or channel. The specificity tests for the used antibodies were performed by incubating other sections in parallel with: (i) PBS instead of the specific primary antibodies and (ii) PBS instead of the secondary antibodies. The results of these controls were always negative (i.e., staining was abolished).

Histometry of the muscle tissue was performed on whole-mount α-actin immunofluorescence larvae. The following parameters were measured: length of five randomly selected myotomes, normalized to the respective larva length; the number of fibers per myotome. These measurements were performed using the ImageJ software (ImageJ 1.53t, NIH, USA, http://imagej.nih.gov.ij/).

#### 2.5.5. Muscle Development—Gene Expression

*2.5.5.1 RNA Extraction and cDNA Synthesis*. Total RNA was extracted from whole larvae using the RNeasy Mini Kit (Qiagen, Hilden, Germany) following manufacturers' instructions. The extracted RNA was eluted in a final volume of 30 μL of Rnase-free water. The concentration and purity of the RNA samples were evaluated with an ND-1000 Nanodrop (Thermo Fisher Scientific, USA). Five hundred nanograms of larvae's RNA were retro-transcribed to cDNA using the Quantitect Reverse Transcription Kit (Qiagen, Hilden, Germany) following the manufacturer's protocol. An additional reaction without the reverse transcriptase was performed to verify the complete genomic DNA removal. Finally, cDNAs were stored at −80°C until subsequent use.

*2.5.5.2 Target Gene Selection and Gene Expression Profiles*. Molecular analyses were conducted to assess the expressions of MRFs. In particular, genes coding for *myod1* and *myf5* (involved in the initial specification of the myogenic lineage), *myog* and *mrf4* (involved in myoblast differentiation), *pax7*, and sox8 (involved in satellite cells maintenance) were selected, as previously described [34–36]. Moreover, β-actin and ef1α were used as reference genes for normalization. Primers' sequences and amplification size of each fragment are described in Table 5. The efficiency and dynamic range of quantitative PCR (qPCR) reactions for target and reference genes were determined using standard curves generated by serial dilutions of samples.

Gene expression was evaluated by qPCR using an iQ5 Real-Time PCR instrument (Bio-Rad, California, USA) and Universal SYBR Green Supermix (Bio-Rad, California, USA) as fluorescent molecules. All reactions were performed in 96 well plates in duplicate in a final 20 μL reaction volume, containing 1 μL of cDNA, 10 μL of 2X SYBR Green and 200 nM (final concentration) of forward and reverse primers for *myf5*, 250 nM for *ef1α*, *myod1*, *myog*, and *pax7*; 400 nM for *sox8*; and 450 nM for *β-actin* and *mrf4*. Annealing temperatures were 60°C for *myod1*, *myf5*, *ef1α*, *myog*, and *sox8*, and 58°C for *mrf-4* and *pax*7 fragment genes. A melting profile was also included. The relative gene expressions of samples were calculated using the *ΔΔ*Ct method, using *β-actin* and *ef1α* as reference genes and samples collected from the control group as reference samples.

### 2.6. Statistical Analyses

For all data concerning reproductive parameters, a one-way ANOVA was executed, with diet (CTR, LI, and HI) as the dependent variable. For the larvae, a two-way ANOVA was applied, with diet (CTR, LI, and HI) and time (hatching and yolk sac absorption) as dependent variables. These analyses of the data were performed with GraphPad Prism software (version 9.5.0). The data are expressed as means ± standard error. Differences between means were considered statistically significant at *p* < 0.05.

## 3. Results

### 3.1. Chemical Composition

The fatty and amino acid profiles of the ingredients are detailed in Table 1. The main fatty acids provided by the ingredients were 16:0 (present in all the ingredients), 16:1*n*−7 (provided mainly *by N. gaditana* biomass), 18:2*n*−6 (present in *H. illucens* and *L. minor*), 18:3*n*−3 (mainly from *L. minor*) and 20:5*n*−3 (provided by *N. gaditana*). Regarding the amino acid content, the contribution of essential amino acids from *L. minor* and *A. esculenta* was lower than *H. illucens* and *N. gaditana* as evidenced by the EAA/NEEAA ratio. The blend of ingredients which is formulated with 85% of these two last ingredients is close to 0.80. Feeds formulated with the ingredients tended slightly to increase the content of 12:0, 18:1*n*−9c, and 20:5*n*−3 when dietary inclusion was increased, but the contribution of the different groups of fatty acids was quite similar among dietary treatments (Table 3). The content of essential amino acids in experimental aquafeeds also slightly varies among treatments, particularly for Leu, Ile in HI compared to CTRL (5.6 vs. 4.2 g 100 g diet; Table 4). The EAA/NEAA ratio ranged from 0.7 to 0.75, being the higher value found in the CTR diet.

### 3.2. Reproductive Parameters

Similar reproductive performance was found among the different groups, both in terms of fecundity (expressed as total eggs per spawn and per kg female) and fertilization (expressed as number of fertilized eggs/spawn/kg female). Fecundity was higher in the CTR group (18901 ± 13) than in the LI (18584 ± 15) or the HI (18621 ± 12); on the opposite, fertilization was lower in the CTR (16066 ± 11) compared with the LI and HI groups (16354 ± 14 and 17318 ± 11, respectively). For both fecundity and fertilization, the differences found between treatments were not statistically significant (*p* > 0.05). Hatching rate and larval survival were significantly higher in the HI group than in the CTR group. No differences were found between the treated groups, LI or HI (Table 6).

### 3.3. Larval Growth

**Figure 1:**
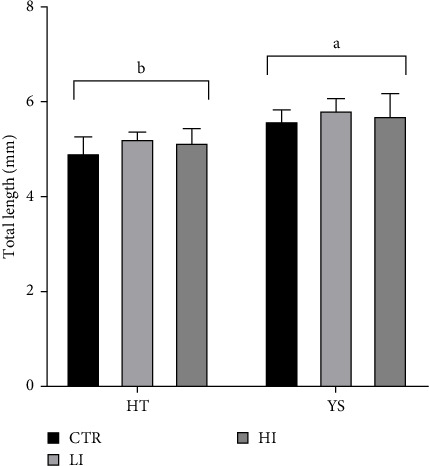
Total length (TL) of larvae at hatch (HT) and at the end of the yolk sac absorption (YS); different letters indicate a significant difference between the two stages (*p* < 0.05). Diet: *p*=0.3108; time: *p* < 0.0006; diet × time: *p*=0.9434. Values are means ± standard error.

The larvae's total length at the yolk sac absorption stage was significantly higher compared to hatching. For each time point, no differences were found between experimental groups (Figure 1, *p* < 0.05).

### 3.4. Skeletal Development

**Figure 2:**
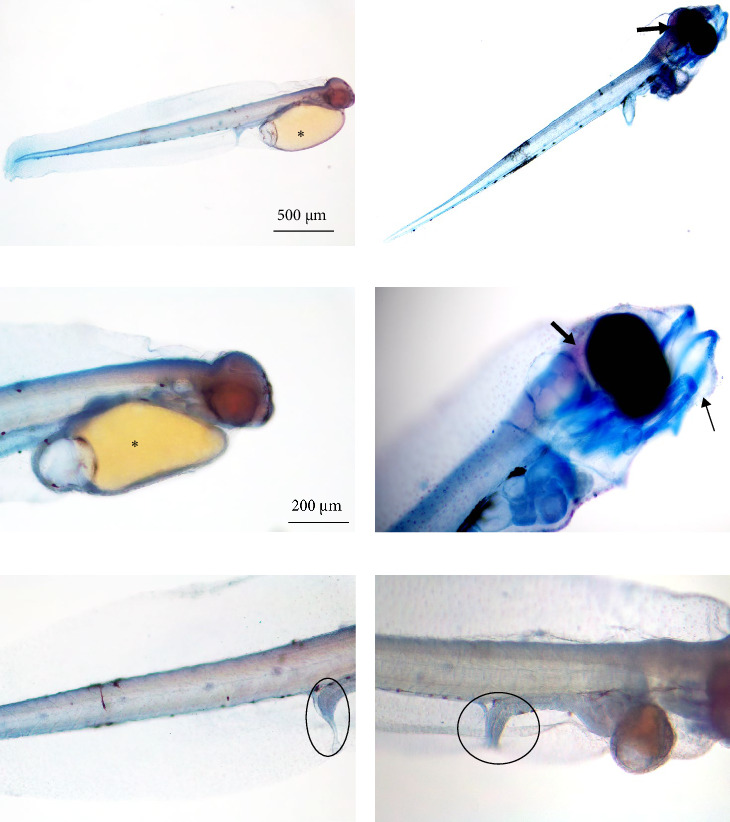
Representative images of seabream larvae at hatching (A, C, E) and the end of the yolk sac absorption (B, D, F). Yolk sac, asterisk; intestine, circle; jaws, thin arrow, ossified neurocranium, bold arrow. Scale bar as located in the images: A,B: 500 µm; C–F: 200 µm.

Alizarin red–Alcian blue wholemount staining showed that larvae at hatching do not yet have an ossified skeleton, as no red staining was detected. At hatching, larvae are still fully cartilaginous (Figure 2A,C,E; blue staining) and no fins are yet developed. Furthermore, it was possible to observe a prominent yolk sac (Figure 2A,C; asterisks), the mouth closed, and a rudimentary intestine (Figure 2E; circle). At the end of the yolk sac absorption, the neurocranium appeared stained in red, therefore, ossified (Figure 2B,D; red color, bold arrow). In contrast, the rest of the body was stained in blue, therefore, cartilaginous. Always at the end of the yolk sac absorption, the pectoral fins were already partially developed, the mouth was already open (Figure 2B,D), and the mandibular bone was formed but still fully cartilaginous (Figure 2D; thin arrow). At this stage, the intestine is still rudimentary but more complex than at hatching (Figure 2F; circle). No differences were found between diets regarding skeletal development.

### 3.5. Muscle Development—Immunofluorescence and Histometry

**Figure 3:**
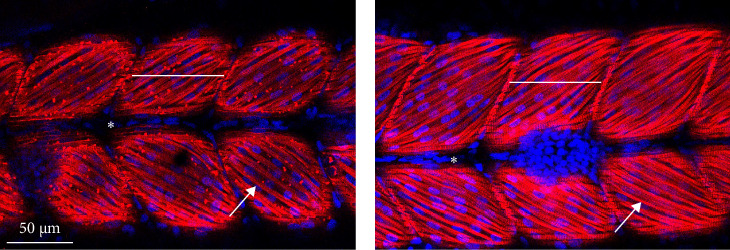
Representative images of alpha-actin-stained larvae at hatching (A) and the end of the yolk sac absorption (B). Myotome, white line; actin myofibril, white arrow; notochord, asterisk. Scale bar as located in (A): 50 µm.

Immunofluorescence with α-actin allowed us to identify the myotomes (Figure 3A,B, white line) with easily distinguishable actin-positive fibers, due to their horizontal and parallel conformation (Figure 3A,B, white arrow).

**Figure 4:**
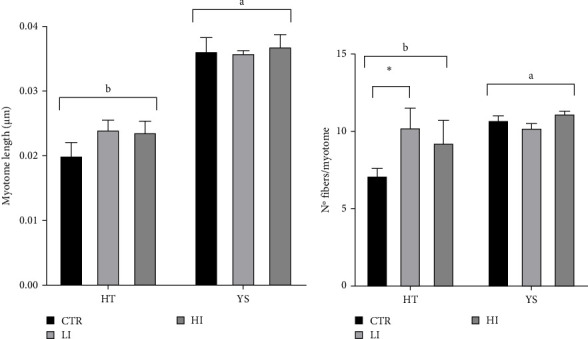
Quantitative representation of the length of the myotome (A) and of the number of fibers per myotome (B) of larvae at hatch (HT) and at the end of the yolk sac absorption (YS). The error bars indicate the standard error of the mean. Asterisks indicate a significant difference between means: *⁣*^*∗*^*p* < 0.05, *⁣*^*∗∗*^*p* < 0.01, *⁣*^*∗∗∗*^*p* < 0.001. Different letters indicate a significant difference between hatching and the end of the yolk sac absorption. Myotome length: Diet: *p*=0.8723; time: *p* < 0.001; diet × time: *p*=0.1030. Number of fibers/myotomes: Diet: *p*=0.2477; time: *p* < 0.0033; diet × time: *p*=0.0076.

Myotome length did not suffer the influence of the diet, but only the influence of time: it was higher at the end of the yolk sac absorption than at hatching (Figure 4A, *p* < 0.001). The same situation was observed concerning the number of fibers per myotome: there was a significant increase in the number of fibers over time (Figure 4B, *p* < 0.01), and the diet per se did not influence this parameter (Figure 4B). There was, however, an interaction between diet and time: at hatching the LI diet showed a significantly higher number of fibers per myotome than the CTR ones (Figure 4B, *p* < 0.05).

### 3.6. Muscle Development—Gene Expression

**Figure 5:**
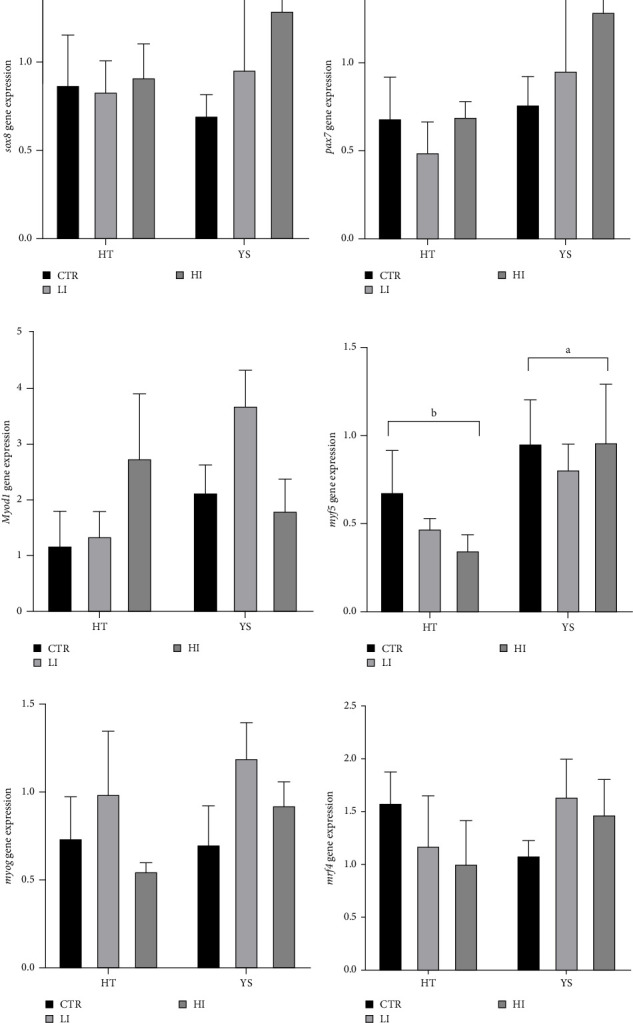
Relative gene expression in larvae at hatch (HT) and at the end of the yolk sac absorption (YS): (A) *sox8*; (B) *pax7;* (C) *myod1*; (D) *myf5*; (E) *myog*; (F) *mrf4*. Different letters indicate a significant difference between hatching and the end of the yolk sac absorption.

Since the analysis related to diet and time did not reveal significant differences, we chose to show the interaction diet × treatment. The relative expression of genes involved in the myogenesis process did not show differences between the three experimental groups (Figure 5), except for *myf5*, which showed a higher expression at the end of the yolk sac absorption than at hatching (Figure 5D, *p* < 0.05).

At hatching, *myf5* and *mrf4* showed the same trend: higher relative expression in animals fed the CTR diet (Figure 5D,F), while the opposite trend was found in the relative expression of *myod1* (Figure 5C). At the end of the yolk-sac absorption, the relative expression of all genes except the *myf5* (Figure 5D) was always lower in animals from the CTR group when compared to both or to one of the supplemented-diet groups (Figure 5).

## 4. Discussion

To fulfill the Sustainable Developmental Goals (SDGs) [38] in terms of the conservation and renewal of marine ecosystems, the growing demand for high-quality food implies innovative production strategies, mainly in terms of novel ingredients [39, 40]. For this reason, the present study takes an important step forward by evaluating the potential of innovative protein sources in supporting sustainable aquaculture practices: four novel ingredients were assessed, and, despite their different chemical composition, experimental diets were formulated to provide similar protein and lipid content (55% and 18%, respectively), as well as amino acid and fatty acid profiles, according to gilthead seabream broodstock's requirements. This approach ensures that the experimental diets closely mirror commercial aquafeeds, allowing for direct comparisons. Commercial aquafeeds for feeding gilthead seabream broodstock are formulated with a nutrient composition close to that used in the present study. For instance, Lancy Breed Essential (Inve Aquaculture) is formulated with 50% crude protein and 15% crude lipid. The consistency in nutrient levels further reinforces the reliability of the observed outcomes, which can be attributed to the novel ingredients themselves.

In fish eggs, protein is the most abundant nutrient and constitutes the main energy source during embryonic development in the majority of the teleost species and plays a major role in fertilization and embryonic development [4]. Its fundamental role highlights the importance of ensuring optimal protein content in broodstock diets, as a key determinant of reproductive success. Indeed, some studies indicate a clear influence of the broodstocks' diet on the total number of spawning eggs per female, fecundity, and oocyte growth and maturation rate, which were shown to be better with higher dietary protein levels in Nile tilapia [41, 42]. A study with European seabass (*D. labrax*) showed a negative effect of a diet with a lower protein content and higher carbohydrate content on egg viability [43]. Low levels of protein in the broodstock diet could cause an alteration of the secretion of GnRHs and LH, known to play an important role in the regulation of oocyte maturation and ovulation, as observed in seabass [4]. These findings support the rationale behind formulating isoproteic diets in the current study, allowing the focus to remain on the specific effects of novel ingredients.

Although fecundity and fertilization appeared to be unaltered by the inclusion of the novel ingredients, the other reproductive parameters were significantly affected. This highlights the need to carefully assess additional variables beyond fecundity and fertilization when evaluating the effects of dietary interventions. Notably, hatching rate and larval survival at the yolk sac absorption stage were higher in the progeny from broodstock fed the diets containing the protein blend. These results suggest potential advantages of the novel protein blend over traditional sources. Although there are few studies on replacement of protein sources in gilthead seabream, there is evidence that the protein source may impact the reproductive parameters. When replacing protein extracted from squid meal with protein from SBM in diets for gilthead seabream broodstock, it was observed a reduction in the hatching rate and larval survival [44]. The authors suggest that this may be due to the SBM composition in terms of fatty acids and phosphorus availability, which are thought to be essential nutrients for reproduction in sparids [45, 46]. Similarly, higher hatching rates were also seen in gilthead seabream when replacing FM with squid meal and the authors claim that this difference could be due to differences in protein apparent digestibility [47]. These observations align with our findings and underscore the hypothesis that the novel protein blend may offer higher digestibility, a hypothesis that deserves further exploration. Another aspect to consider is the potential impact of soy-derived isoflavones on reproductive performance. Soybeans contain large amounts of isoflavones, which are phenolic compounds that are known to bind to fish estrogen receptors [48] and that may cause detrimental effects on the reproductive performance [49]. However, the use of SPC in this study minimizes the potential influence of isoflavones, as SPC contains significantly lower levels of these compounds compared to SBM [50]. Thus, while the isoflavone content in the diets is unlikely to explain the observed differences, additional studies are needed to confirm these findings.

Regarding larval development, it is important to note that the total length of larvae at early developmental stages has not been studied in depth. Most studies focus on stages following mouth opening and exogenous feeding. For instance, authors report an average length at hatching of around 2.5–3 mm and an average length at the beginning of exogenous feeding of approximately 4 mm [4, 51]. However, these data are not consistent with the measurements from this study, where the average length at T0 was 5 and 6 mm at T1. This discrepancy can be explained by the selection of broodstocks used in this study. The broodstocks were thoroughly chosen, with females identified as well-seasoned breeders. These findings are consistent with studies showing that broodstock selected for high growth traits produce larvae with larger sizes at early stages in gilthead seabream by http://10.1016/j.anireprosci.2022.106989. However, the length of the larvae of all three dietary groups showed the same physiological trend observed in the literature [52].

Moving from overall larval length to skeletal development, we observe another crucial aspect of early fish growth. The first skeletal structures in fish appear after hatching and, therefore, only the most severe deformities are visible at the stage of growth analyzed in this study. The majority of the deformities are only observed in the juvenile phase, when the skeletal system is developed enough and can reach 80% [53]. The results obtained regarding skeletal development were, for the most part, consistent with what was reported in many studies. The three studies carried out by Faustino and Power [51, 54, 55], constitute the most accurate and detailed studies on larvae of gilthead seabream, where the whole process of cartilage and bone skeletal development of all the structures that make up the fish skeleton are thoroughly analyzed. In our study, at T0, we did not observe cartilaginous or bone structures, which is in line with what was described by Faustino and Power [51] and Saka et al. [56]. At T1, the jaws showed cartilaginous structures, which were also by Faustino and Power [51] and Saka et al. [56] at the same developmental stage. On the contrary, Riera-Heredia et al. [57] found that the skeleton of the jaw and the mouth were formed only after the onset of the exogenous feeding. Always at the end of the yolk sac absorption, Faustino and Power [51] observed that many other bones in the head were developing, such as the ones forming the viscerocranium and the neurocranium. In this study, the development of the viscerocranium and the neurocranium was not observed, probably because our study lasted until the end of the endogenous feeding phase (T1), while the other studies continued the observations [51, 54–56]. The beginning of exogenous feeding is a crucial stage for many physiological processes, and therefore, some results could not be observed in our study. It has been postulated that the early development of the head bone structures, especially the jaws, is strongly related to the onset of exogenous feeding and the need to hunt for food. Having examined the skeletal development, the discussion transitions to the impact of diet on these early stages. No differences could be found in the skeletal development of larvae of the three dietary groups, so we can state that using the protein blend in the broodstock diets does not impact the skeletal development of the larvae.

From skeletal development, attention shifts to muscle growth, another vital aspect of larval development. In gilthead seabream, from hatching to the yolk sac absorption, the development of the muscles occurs through stratified hyperplasia, while hypertrophy only starts at 35 days posthatching [58, 59]. It was, therefore, expected to observe an increase in the number of fibers, as well as an increase in their length, as the larvae grew from T0 to T1. The technique used for this process allowed us to closely monitor the muscle's development and identify eventual differences in the different experimental groups. No differences in the myotome length were found among the larvae at the same growth stage, indicating that the diet does not have an impact on their development. The only difference observed in the myotome length was between T0 and T1 larvae, which is an expected result since the larvae undergo an overall substantial growth in the period of time from hatching to yolk sac absorption. Since the muscle tissue is essential for movement, it is necessary that this is developed and able to support body movements when exogenous feeding starts, and the larvae need to search for food. The number of fibers also shows a significant difference over time, and this is consistent with the expected hyperplastic development of the muscles. Interestingly, at T0, larvae from the CTR diet had significantly fewer fibers compared to the LI group, though this difference was not found at the end of the trial.

Finally, gene expression patterns offer another dimension to understanding muscle differentiation and growth.

In vertebrates, MRFs (*myod1*, *myf5*, *myogenin*, and *mrf4*) are sequentially expressed during myogenesis: *myod1* and *myf5* are involved in the initial specification of the myogenic lineage, while *myogenin* and *mrf4* are linked to myoblast differentiation [34–36].

Studies on in vitro cultured gilthead seabream cells showed a peak in *myf5* expression at Day 2, followed by a decrease, whereas *myogenin* and *mrf4* exhibited low expression until Day 7, peaking at Day 8 [60]. It was hypothesized that MRF expression follows a similar pattern: higher *myod1* and *myf5* at T0, and lower at T1, with the opposite trend for *myogenin* and *mrf4*. However, our results do not align with this pattern: no difference in the gene expression of the four genes analyzed at T0 and T1, except for *myf5*, which seems to have a slight increase in expression from T0 to T1, which is the opposite of what is found in the literature. This may indicate increased recruitment of myoblasts, which fuse to form new fibers or are incorporated into existing fibers during hypertrophic growth, according to Watabe [61]. This result aligns with the observed larval growth and histometric data. Overall, the replacement of soy with the protein blend did not affect skeletal or muscle development or growth potential between diets.

## 5. Conclusions

The main issue for carnivorous species, particularly broodstock, is the proteinaceous alternative ingredients needed to fulfill the requirements without producing negative impacts. Specifically, this study highlighted the potential of alternative protein sources for gilthead seabream, demonstrating that a novel blend of ingredients may assist reproductive performance and offspring development. Including insect meal, duckweed, microalgae, and seaweed in the broodstock diet maintained larval growth and muscle development and enhanced key reproductive parameters such as hatching rates and larval survival during the yolk sac absorption. These findings are promising for the development of a sustainable aquaculture, suggesting that traditional protein sources, such as soy in broodstock feeds, can be partially replaced without compromising performance or offspring quality. However, this research focused on short-term effects in larvae, and further studies are needed to investigate potential long-term implications of these diets on fish health and productivity. Such dietary innovations may benefit commercial hatcheries by reducing dependency on conventional feed ingredients while maintaining high biological and economic efficiency.

## Figures and Tables

**Table 1:** Fatty acid (% total fatty acids) and amino acid (g/100 g dry basis) profiles of the alternative ingredients and the blend used in the elaboration of experimental feeds.

Fatty acid (%)	*Hermetia illucens*	*Lemna minor*	*Nannochloropsis gaditana*	*Alaria esculenta*
C12:0	9.3	—	—	—
14:0	8.2	2.6	3.2	5.0
16:0	16.5	23.9	18.8	15.7
16:1*n*−7	3.1	2.4	25.0	1.6
16:2*n*−4	0.2	—	1.9	—
18:0	3.5	3.6	0.7	1.8
18:1*n*−9	14.8	2.8	1.4	11.2
18:1*n*−7	—	2.8	0.5	—
18:2*n*−6	17.3	11.7	2.4	5.0
18:3*n*−3	1.4	27.4	0.9	11.3
18:4*n*−3	1.1	4.0	0.4	18.4
20:4*n*−6	—	—	0.5	—
20:4*n*−3	—	—	2.1	14.4
20:5*n*−3, EPA	—	2.4	22.6	—
22:5*n*−3	—	3.0	—	—
Amino acid (g/100 g)	—	—	—	—
*EAA*				
Arg	2.8	1.4	2.1	0.4
His	1.4	0.4	0.4	0.4
Leu	3.8	1.8	2.8	0.7
Ile	2.5	1.0	1.4	0.4
Lys	4.2	1.7	2.5	0.7
Met	1.0	0.5	0.6	0.2
Phe	2.4	1.2	1.6	0.5
Thr	2.1	1.1	1.6	0.4
Val	3.8	1.9	1.8	0.5
*NEAA*				
Ala	4.7	1.6	3.0	0.9
Asp	5.1	2.4	3.0	1.0
Cys	0.3	0.3	0.1	0.0
Glu	6.2	2.6	4.5	1.1
Gly	3.1	1.3	1.7	0.5
Pro	2.7	10.1	3.2	1.5
Ser	2.3	1.1	1.4	0.5
Tyr	4.3	0.9	1.1	0.4
*EAA/NEAA*	0.8	0.5	0.8	0.7

*Note:* Cysteine and methionine suffer transformation during acid hydrolysis, as cysteine can be destroyed while methionine can be oxidized. Tryptophan is destroyed during acid hydrolysis. Serine and threonine are partially destroyed during acid hydrolysis.

Abbreviations: EAA, essential amino acid; NEAA, non-EAA.

**Table 2:** Ingredient and chemical composition (g/100 g dry basis) of the experimental diets used in the present study.

Ingredient (g/100 g dry basis)	CTR	LI	HI
Fishmeal LT94^a^	40.00	40.00	40.00
Insect meal^b^	—	2.00	4.00
Duckweed meal^c^	—	0.50	1.00
*Nannochloropsis gaditana* meal^d^	—	2.25	4.50
*Alaria esculenta* meal^e^	—	0.25	0.50
Squid meal^f^	5.00	5.00	5.00
CPSP90^g^	3.00	3.00	3.00
Krill meal^h^	10.00	10.00	10.00
Wheat gluten^i^	11.00	11.00	11.00
Soybean protein concentrate^j^	6.50	3.50	0.30
Yeast^k^	0.20	0.20	0.20
Fish oil^l^	6.10	6.10	5.70
DHA + EPA oil^m^	2.00	2.00	2.00
Krill phospholipids^n^	1.00	1.00	1.00
Astaxanthin^o^	0.02	0.02	0.02
Wheat meal^p^	12.00	10.00	8.60
Choline chloride^q^	0.24	0.24	0.24
Betaine^r^	0.24	0.24	0.24
Monosodium phosphate^s^	1.00	1.00	1.00
Vitamin and mineral premix^t^	1.50	1.50	1.50
Vitamin C^u^	0.10	0.10	0.10
Vitamin E^v^	0.10	0.10	0.10
Crude protein (% dry matter)	55.6	55.0	54.1
Crude lipid (% dry matter)	17.6	18.0	18.5
Ash (% dry matter)	9.2	9.5	10.5
Moisture (%)	7.6	8.3	7.6
Nfe^w^	17.6	17.5	16.9
Estimated gross energy (MJ/kg)^x^	18.88	18.92	18.85

*Note:* Dietary codes: CTR: control diet without alternative ingredients. HI, diet containing 10% of the alternative ingredient blend; LI, diet containing 5% of the alternative ingredient blend.

^a^69.4% crude protein, 12.3% crude lipid (Norsildemel, Bergen, Norway).

^b^56.5% crude protein, 15.2% crude lipid (ATB—Leibniz-Institut fur Agrartechnik, Potsdam, Germany).

^c^33.5% crude protein, 6.6% crude lipid (Bantry Marine Research Station, Bantry, Ireland).

^d^31.4% crude protein, 21.3% crude lipid (University of Almería, Spain).

^e^17.2% crude protein, 8.6% crude lipid (Institute of Technology of Agricultural Products, Greece).

^f,g,h^Purchased from Bacarel (UK). CPSP90 is enzymatically pre-digested fishmeal.

^i^78% crude protein (Lorca Nutrición Animal SA, Murcia, Spain). Total P: 0.718 g 100 g; total phytate P: 0.01 g 100 g.

^j^Soycomil, 60% crude protein, 1.5% crude lipid (ADM, Poland). Total P: 0.66 g 100 g; total phytate P: 0.42 g 100 g.

^k^Local provider (Almeria, Spain).

^l^AF117DHA (Afamsa, Spain).

^m^Veramaris oil (500 mg DHA + 100 mg EPA/g).

^n^350 mg phospholipids/g, (QRILL AQUA, Aker BioMarine Antarctic AS (Norway).

^o^ *Haematococcus pluvialis* (Necton, Portugal).

^p,q,r,s^Lorca Nutrición Animal SA (Murcia, Spain).

^t^ *Lifebioencapsulation* SL (Almería, Spain). Vitamins (mg kg^−1^): vitamin A (retinyl acetate), 2,000,000 UI; vitamin D3 (DL-cholecalciferol), 200,000 UI; vitamin E (Lutavit E50), 10,000 mg; vitamin K3 (menadione sodium bisulfite), 2500 mg; vitamin B1 (thiamin hydrochloride), 3000 mg; vitamin B2 (riboflavin), 3000 mg; calcium pantothenate, 10,000 mg; nicotinic acid, 20,000 mg; vitamin B6 (pyridoxine hydrochloride), 2000 mg; vitamin B9 (folic acid), 1500 mg; vitamin B12 (cyanocobalamin), 10 mg vitamin H (biotin), 300 mg; inositol, 50,000 mg; betaine (Betafin S1), 50,000 mg. Minerals (mg kg^−1^): Co (cobalt carbonate), 65 mg; Cu (cupric sulfate), 900 mg; Fe (iron sulfate), 600 mg; I (potassium iodide), 50 mg; Mn (manganese oxide), 960 mg; Se (sodium selenite), 1 mg; Zn (zinc sulfate) 750 mg; Ca (calcium carbonate), 18.6%; (186,000 mg); KCl, 2.41%; (24,100 mg); NaCl, 4.0% (40,000 mg).

^u,v^TECNOVIT, Spain.

^w^Nitrogen free extract (Nfe) = 100 − (crude protein + crude lipid + ash).

^x^The total caloric content (MJ/kg) was estimated using the commonly accepted energy coefficients for the major chemical components: protein (16,74), oil (37.66), and carbohydrates (16.74) [29].

**Table 3:** Fatty acid content of the experimental diets (g/100 g feed dry basis).

Fatty acid	CTRL	LI	HI
C12:0	0.02	0.17	0.28
C14:0	1.06	1.06	1.06
C14:1	0.02	0.01	0.02
C15:0	0.12	0.12	0.12
C16:0	3.98	3.84	3.92
C16:1*n*−7	0.91	0.94	0.96
C17:0	0.12	0.12	0.13
C18:0	0.74	0.73	0.79
C18:1*n*−9t	0.02	0.02	0.02
C18:1*n*−9c	2.11	2.13	2.21
C18:2*n*−6t	0.03	0.02	0.02
C18:2*n*−6c	0.86	0.84	0.83
C18-3*n*−6	0.03	0.04	0.05
C20:0	0.05	0.05	0.06
C18:3*n*−3	0.23	0.24	0.23
C20:1*n*−9	0.26	0.26	0.27
C20:2*n*−6	0.05	0.05	0.05
C20:3*n*−6c	0.02	0.02	0.02
C22:0	0.07	0.03	0.05
C22:1*n*−9	0.05	0.04	0.04
C20:4*n*−6	0.02	0.01	0.02
C23:0	0.20	0.22	0.24
C20:5*n*−3, EPA	1.49	1.65	1.67
C24:0	0.03	0.03	0.03
C24:1	0.07	0.08	0.09
C22:6*n*−3, DHA	3.62	3.34	3.6
*Σ*SFA (%)	39.49	39.66	39.81
*Σ*MUFA (%)	15.64	15.82	15.79
*Σ*PUFA (%)	39.25	38.67	38.68
*Σn*−3 (%)	33.00	32.57	32.78
*Σn*−6 (%)	6.12	5.98	5.78
*Σn*−9 (%)	15.08	15.26	15.14
*Σn*−3 PUFA (%)	33.00	32.57	32.78
*n*−3/*n*−6	5.39	5.45	5.67
EPA/DHA	0.41	0.49	0.46

*Note:* Dietary codes: CTR: control diet without alternative ingredients. HI, diet containing 10% of the alternative ingredient blend; LI, diet containing 5% of the alternative ingredient blend.

Abbreviations: DHA, docosahexaenoic acid; EPA, eicosapentaenoic acid; MUFA, monounsaturated fatty acid; PUFA, polyunsaturated fatty acid; SFA, saturated fatty acid.

**Table 4:** Amino acid profile (g/100 g feed dry basis) of the experimental diets.

Amino acids	CTRL	LI	HI
EAA			
Arg	4.1	4.0	3.9
His	1.1	1.1	1.0
Leu + Ile	5.6	4.8	4.2
Lys	2.6	2.3	2.3
Met	0.6	0.7	0.7
Phe	2.3	2	1.9
Thr	1.6	1.5	1.3
Val	2.0	1.7	1.7
NEAA			
Ala	2.5	2.6	2.4
Asx	4.0	4.2	3.6
Cys	0.3	0.3	0.3
Glx	12.5	12.2	12.2
Gly	1.9	1.7	1.6
Pro	1.3	1.3	1.1
Ser	2.7	2.6	2.3
Tyr	1.2	1.0	0.9
EAA/NEAA	0.75	0.70	0.70

*Note:* Cysteine and methionine suffer transformation during acid hydrolysis, as cysteine can be destroyed while methionine can be oxidized. Tryptophan is destroyed during acid hydrolysis. Serine and threonine are partially destroyed during acid hydrolysis. Dietary codes: CTR, control diet without alternative ingredients; HI, diet containing 10% of the alternative ingredient blend; LI, diet containing 5% of the alternative ingredient blend.

Abbreviations: EAA, essential amino acid; NEAA, non-EAA.

**Table 5:** qPCR primer sequences and amplification product lengths.

Gene	Primer forward (5′–3′)	Primer reverse (5′–3′)	Size (bp)	Reference
*β-actin*	TCCTGCGGAATCCATGAGA	GACGTCGCACTTCATGATGCT	51	[37]
*EF1α*	CTTCAACGCTCAGGTCATCAT	GCACAGCGAAACGACCAAGGGGA	263	[37]
*myf5*	CTACGAGAGCAGGTGGAGAACT	TGTCTTATCGCCCAAAGTGTC	183	[34]
*mrf4*	CATCCCACAGCTTTAAAGGCA	GAGGACGCCGAAGATTCACT	151	[34]
*Myog*	CAGAGGCTGCCCAAGGTGGAG	CAGGTGCTGCCCGA ACTGGGCTC	182	[37]
*myod1*	GTTTTGTTCCAGGCGGTCT	GCTGGTGTCGGTGGAGAT	98	[37]
*pax7*	ATGAACACTGTCGGCAACG	AGGCTGTCCACACTCTTGATG	185	[34]
*sox8*	TCTCAGGTCCTCAAGGGATACG	GCCCAAACCATGAACGCAT	116	[34]

**Table 6:** Egg and larval quality in the three experimental groups.

Reproductive parameters	CTR	LI	HI
Hatching rate (%)	86 ± 1.74^a^	87 ± 1.63^ab^	91 ± 1.21^b^
Larval survival (%)	73 ± 2.92^a^	78 ± 2.42^b^	78 ± 2.92^b^

*Note*: Within a row means with different superscript letters differ significantly (*p* < 0.05). Values are means ± standard error.

## Data Availability

All the data used to support the findings of this study are included in this paper.
